# Is There a Difference in Immune Response to SARS-CoV-2 Vaccination between Liver and Lung Transplant Patients with Cystic Fibrosis?

**DOI:** 10.3390/vaccines11030657

**Published:** 2023-03-14

**Authors:** Teresa Fuchs, Dorothea Appelt, Helmut Ellemunter

**Affiliations:** Department of Child and Adolescent Health, Paediatrics III, Cystic Fibrosis Centre Innsbruck, Medical University of Innsbruck, 6020 Innsbruck, Austria

**Keywords:** Cystic Fibrosis, COVID-19, mRNA vaccine, transplantation

## Abstract

People with Cystic Fibrosis (CF), especially solid organ transplant recipients, have been prioritized in the SARS-CoV-2 vaccination program. This study assesses antibody response of patients with CF who have undergone liver (CF-LI) or lung (CF-LU) transplantation, and compares results to published data of patients with solid organ transplantation without CF as underlying disease. Antibodies against the spike receptor-binding domain were measured within the routine visits at the CF Centre in Innsbruck, Austria, after the second and third doses of SARS-CoV-2 mRNA vaccines. We report on 13 adult CF patients who are recipients of solid organ transplant, including five CF-LI and eight CF-LU. Overall, 69% had measurable antibody response after two, and 83% after three doses of SARS-CoV-2 vaccines. In CF-LI, positive serological response amounted to 100% after two and three doses, while CF-LU showed only a 50% and a 71% response rate, respectively. Clear differences are seen between the CF-LI and CF-LU groups in our cohort, with worse response rate for lung transplant recipients. Immune response between CF-LI and CF-LU, therefore, must be considered in a differentiated manner, and the importance of booster vaccination is once more emphasized with these data.

## 1. Introduction

Cystic Fibrosis (CF) is a congenital multisystem disorder caused by mutation in the CF transmembrane conductance regulator (CFTR) gene. Mutations in the CFTR gene cause loss of function of the CFTR protein, which normally transports chloride and bicarbonate. This results in the classic symptoms of CF including chronic pulmonary infections, progressive inflammatory processes, endocrine pancreatic insufficiency, hepatopathy, and numerous other comorbidities [1]. Although CF affects many organs, morbidity and mortality are mainly determined by pulmonary involvement. A vicious cycle of inflammation and infection is described, with uncertainty about which process occurs first. This leads to progressive destruction of lung tissue with development of bronchiectasis [2]. Lung transplantation is still considered the ultimate therapeutic option in individuals with advanced CF lung disease, despite novel CFTR therapy [3]. CF liver disease (CFLD) is the second leading cause of death for individuals with CF, with an incidence of approximately 25%. End-stage CFLD includes cirrhosis with portal hypertension leading to liver transplantation as the only potentially curative treatment [4].

### Cystic Fibrosis and SARS-CoV-2

The novel coronavirus SARS-CoV-2 was identified in Wuhan, China, in December 2019 and declared a global pandemic in March 2020 by the World Health Organization (WHO). Since then, SARS-CoV-2 disease, also called COVID-19, has spread worldwide causing more than 660 Mio infections and over 6.7 Mio deaths [5]. It is known that viral infections, such as influenza virus, can cause pulmonary exacerbations in patients with chronic lung disease. Consequently, individuals with CF have quickly been identified as a risk group for severe COVID-19 disease [6]. Outcome data from patients with CF who developed COVID-19 were rapidly published, showing a better experience than originally feared [7]. General findings of cumulative data reveal that people with CF are as vulnerable to infection as the general population [8]. According to Jung et al., who studied data on 828 infected CF patients in the European Cystic Fibrosis Society Patient Registry, general symptoms were the most common, followed by fever, increased cough, fatigue, myalgia/arthralgia, and pulmonary exacerbation [9]. Nevertheless, factors for severe outcomes in subgroups following COVID-19 infection have been described. Regarding pulmonary colonization status, *Pseudomonas aeruginosa* (PA) and *Achromobacter* spp. have been associated with increased risk of hospitalization [9]. One of the most relevant predictors of severe disease is the presence of a severely deteriorated lung function before infection, especially when presenting with FEV_1_ < 40%. Previous transplant status is also considered one of the main risk factors for severe outcome, with higher risk of hospitalization, respiratory support, admission in an ICU, or death [10]. Therefore, people with CF, especially solid organ transplant recipients, have been prioritized in the SARS-CoV-2 vaccination program as a potential clinically fragile patient population. In Austria, the national vaccination board recommended additional vaccination to basic immunization (third dose of vaccine) for patients who are immunocompromised, including history of transplantation [11]. Studies have shown low antibody response to SARS-CoV-2 vaccines in recipients of solid organ transplant compared to healthy population. Risk factors that lead to a decreased immune response after two vaccinations have been described and include older age, recent transplantation status, receipt of organ transplants from deceased donor, and active use of antimetabolite immunosuppression (mycophenolate mofetil, mycophenolic acid, azathioprine) [12]. Although improvements have been documented in antibody response after dose three compared to dose two, such results are still low compared to non-transplanted patients [12,13,14]. Factors negatively influencing antibody response include higher daily dose of mycophenolic acid and/or immunosuppressive combination therapy in liver transplantation [13], older age, and receipt of an organ from a deceased donor [12]. Factors associated with a better serologic response include contracting COVID-19 prior to vaccination, higher IgG values, and male sex [12,14].

Data regarding immune response to SARS-CoV-2 mRNA vaccination in CF patients are limited, especially data on solid organ transplant recipients. In this study, we assess antibody response of patients with CF who have undergone liver (CF-LI) or lung (CF-LU) transplantation, and compare results to recipients of solid organ transplant without CF as underlying disease. The purpose of the study is to expand knowledge of the efficacy of vaccination in transplanted CF patients.

## 2. Methods

Antibody response was measured during routine visits at the CF Centre in Innsbruck, Austria. Patients routinely visit the CF Centre at least four times a year. Among several examinations, lung function test and extensive laboratory analyses are routinely performed, including SARS-CoV-2 antibodies (regardless of vaccination status). We started the determination of SARS-CoV-2 antibodies in fall of 2020. In this study, antibodies against the receptor-binding domain of S1 subunit of spike protein (S1RBD) were used as laboratory values. Measurement of antibodies were performed using chemiluminescent microparticle immunoassay. Positive cut off was set at 7.0 BAU/mL [15]. Data are presented using median and range for metric variables and absolute/relative frequencies for categorical data. The exact binomial test was used to calculate the 95% confidence intervals for the data comparisons. Statistical analysis was performed using R statistical software (Foundation for Statistical Computing, Vienna, Austria 2022).

## 3. Results

### 3.1. Patient Characteristics

The CF Centre Innsbruck treats a total of 200 mixed paediatric and adult patients. Of these patients, 14 received an organ donation. One patient aged two years with a history of liver transplantation was excluded from the study, as he was the only paediatric participant without the possibility of SARS-CoV-2 vaccination for his age group at that time in Austria. The other 13 adult recipients of solid organ transplants had been vaccinated at least twice with a SARS-CoV-2 mRNA vaccine (30 µg Comirnaty and/or 50 µg Spikevax). All 13 patients were included in this real-world cohort study, including five CF-LI and eight CF-LU (among them, one with lung and kidney transplantation). In total, there were eight male and five female participants (CF-LI: four males, one female; CF-LU: four males, four females). Patients were generally younger in the CF-LI group, with an average age of 21 years (range 20–46 years) compared to 41 years in the CF-LU group (range 32–61 years). Mean time interval since performance of transplantation was 11 years in CF-LI (range 9–22 years) and 10 years in CF-LU (range 6–24 years). 

Lung function showed moderate to normal values in both groups, with 78% ppFEV1 in CF-LI (range 42–82%) compared to 81% (range 49–118%) in CF-LU after the third vaccination according to global lung initiative [16]. BMI status was comparable in both groups, with 20 kg/m^2^ in CF-LU and 22 kg/m^2^ in CF-LI. In CF-LI, three out of five patients were treated with mycophenolic acid in combination with tacrolimus or everolimus, no patient was taking cortisone regularly, and one patient was treated with azithromycin. In CF-LU, all eight patients received combination immunotherapy (five received tacrolimus/mycophenolic acid and three received tacrolimus/everolimus) and permanent treatment with cortisone. Regarding long time antibiotic therapy, four patients were treated with trimethoprim and one with sulfamethoxazole. 

Regarding chronic bacterial lung colonisation, seven patients in total were classified as chronically PA infected according to modified Leeds criteria (three in CF-LI, four in CF-LU). All patients with chronic PA infection and CF-LI were on long-term inhaled antibiotic therapy. Achromobacter was not detected by sputum culture in any patient. Patient characteristics are shown in Table 1.

After receiving the second and third anti-SARS-CoV-2 vaccination, no side effects such as fever, asthenia, or myalgia were reported. In CF-LU, two patients contracted COVID-19 between administration of the second and third vaccinations. One participant required hospitalization due to decreased general condition. A third patient was diagnosed with COVID-19 after the third vaccination with need for intensive care due to pre-renal kidney failure. No patient died from COVID-19, although one participant died due to reasons unrelated to COVID-19 infection or SARS-CoV-2 vaccination.

### 3.2. Monitoring Secreted Antibodies against SARS-CoV-2

Antibodies were measured after the second and third dose of SARS-CoV-2 mRNA vaccine, 47 and 63 days, on average, after the two vaccinations, respectively. Three patients in the CF-LU group suffered from Covid-19 infection before receiving the third vaccination.

Overall, 69% had a measurable antibody response higher than the cut off after two doses, with CF-LI showing a 100% positive serological response and CF-LU only 50%. After the third dose of the vaccine, positive serological response rate again scored 100% in CF-LI, 71% in CF-LU, and 83% overall. 

**Figure 1:**
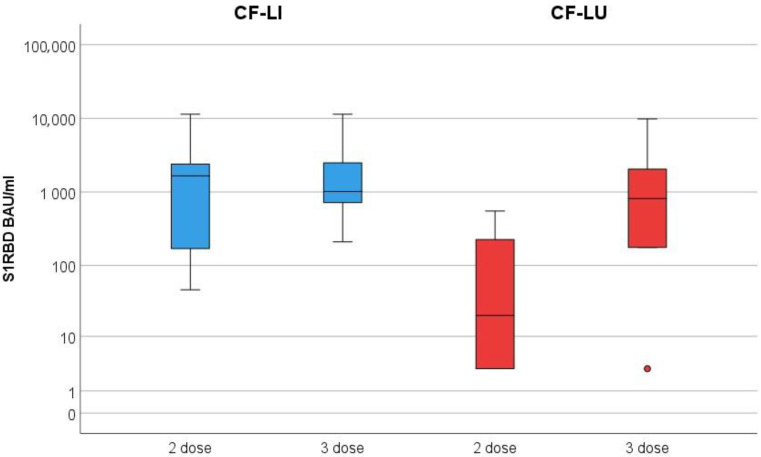
Comparison of S1RBD levels after two and three doses between CF-LI (in blue) and CF-LU (in red) showing higher antibody response in CF-LI after two and three doses of the vaccine, respectively.

The median S1RBD antibody level after dose two in CF-LI was 3124.2 BAU/mL (range 46–11,360) compared to 105 BAU/mL (range <3–551.9) in CF-LU. After dose three, the median S1RBD antibody level in CF-LI was 3158.1 BAU/mL (range 209.4–11,360) compared to 2000 BAU/mL (range <3–9815.2) in CF-LU. Changes in S1RBD antibodies are shown in Figure 1.

## 4. Discussion

In this study, the majority of patients had a detectable antibody response after two and especially after three doses of SARS-CoV-2 mRNA vaccine. The CF-LI group showed a 100% response rate after the second and third vaccination. An Italian study group enrolled 107 patients in a cohort study 91 months, on average, after performed liver transplantation. They were vaccinated with BNT16b2 vaccine. Six months after the second dose of the vaccine, 76.6% of patients showed positive antibody response compared to 100% in our patient population. After the third dose, 91.6% developed a positive response, again compared to 100% in our data [13]. Therefore, all of our patients who received liver transplantation showed an antibody response after the second vaccination and continued to show elevated antibody levels after the third dose. The proportion of nonresponding patients, as shown in multiple other studies, is not confirmed in our data, although the small patient population must be emphasized as a disadvantage [17].

The CF-LU group showed a 50% positive response rate after two doses and a 71% response after three vaccinations. Comparing the results to data from Hoffman et al., who studied 89 patients with lung transplantation from the Netherlands (CF patients excluded), our data show generally higher rates (50% vs. 35% after second and 71% vs. 62% after third vaccination). Patients in this study were vaccinated with BNT162b2 or mRNA-1273 [14]. A factor associated with a better serological response after the third vaccination in our cohort was found to be contracting a COVID-19 infection prior to the third dose (two patients). This factor is also described in the study by Hoffman et al. [14]. After receiving the third vaccination, one of our patients contracted COVID-19 during the observational period. This suggests that patients who have undergone lung transplantation remain at risk of developing COVID-19 despite two or three vaccinations.

Our data from the CF-LU group show a comparable response rate to a meta-analysis by Manothummetha et al., matching the already existing data for patients with solid organ transplantation without CF (P = 1.00). Eighty-three studies were included in this systemic review and 29 studies were included in the meta-analysis, representing 11,713 recipients of solid organ transplants. Overall, these data show low antibody response despite multiple doses of mRNA vaccines [12]. 

In our cohort, clear differences in antibody response can be seen between CF-LI and CF-LU, with a worse response rate in lung transplant recipients although this group also involves patients who have experienced COVID-19 infection.

Toniutto et al. describes high daily doses of mycophenolic acid and/or immunosuppressive combination therapy as the main risk factors for negative antibody response in liver transplant recipients [13]. Three out of five CF-LI patients in our cohort were treated with mycophenolic acid in combination with tacrolimus or everolimus and still showed a 100% response rate. Likewise, other studies showed no correlation between negative serologic response rate and mycophenolic acid therapy [12,14]. Possible reasons for different antibody reactions after vaccination between CF-LI and CF-LU could not be investigated statistically in the present study due to the small patient population. However, worse serologic response in CF-LI compared to other solid organ transplant recipients has already been described in other studies. Factors associated with higher antibody values include younger age, normal response to previous pneumococcal vaccination, and contraction of COVID-19 infection before vaccination [12,14]. Comparison of the mean age (34 years) within our study with other study populations shows clear differences, with our cohort being generally younger [13,14]. Within our patient population, CF-LI patients were significantly younger compared to CF-LU (21 years vs. 41 years). It should also be emphasized that, in our data, CF-LU, in contrast to CF-LI patients, received higher doses of immunosuppression, including permanent therapy with cortisone.

In summary, these data demonstrate the antibody response to SARS-CoV-2 vaccination in patients with CF and solid organ transplantation. The serologic response improved after a third vaccine dose. A major limitation of the study is the small population size, which mostly allows descriptive statistics, the single centre study format, and the lack of a control group. We also mainly focussed on serologic response to vaccination, although there is an urgent need to evaluate parameters correlating the level of antibody levels with the extent of clinical symptoms during COVID-19 infection. Our data add new information to the sparse data available so far on this specific vulnerable patient population, and suggests that the immune response of patients with CF and organ transplantation is comparable to those without CF; our data may even indicate a better response. Our data could be compared with data on antibody levels in CF patients without transplantation, which tend to show higher levels compared to a healthy (but older) control group [18].

In conclusion, immune response between CF-LI and CF-LU must be considered in a differentiated manner, and the importance of booster vaccination is once more emphasized.

## Figures and Tables

**Table 1:** Characteristics of participants.

Item	Total	Liver	Lung
(N)	13	5	8
Sex n (%)			
male	8 (62%)	4 (80%)	4 (50%)
female	5 (38%)	1 (20%)	4 (50%)
Age (y) median (range)	34 (20, 61)	21 (20, 46)	41 (32, 61)
time since Tx (y) median (range)	11 (6, 24)	11 (9, 22)	10 (6, 24)
FEV1% median (range)		78 (42, 82)	81 (49, 118)
LCI median (range)		12.6 (8.8–16.6)	9 (7.2–12)
BMI/kg/m^2^) median (range)		20 (19, 22)	22 (16, 26)
CFRD n (%)	10 (77%)	4 (80%)	6 (75%)
Insulin therapy	9 (69%)	3 (60%)	6 (75%)
Mycophenolic acid therapy n (%)	11 (84%)	3 (60%)	8 (100%)
Azithromycin therapy n (%)	1 (7%)	1 (20%)	0 (0%)
Chron. PA colonisation n (%)	7 (54%)	3 (60%)	4 (50%)
Serologic Response n (%)			
positive 2. Vaccine	9 (69%)	5 (100%)	4 (50%)
(N) *	12	5	7
positive 3. vaccine	10 (83%)	5 (100%)	5 (71%)

* One patient died before receiving the third vaccination, due to causes unrelated to COVID-19 vaccination.

## Data Availability

The data presented in this study are available on request from the corresponding author. The data are not publicly available due to privacy and ethical reasons.

## Abbreviations

CF	Cystic Fibrosis
CFTR	CF transmembrane conductance regulator
CFLD	CF liver disease
SARS-CoV-2	Severe acute respiratory syndrome coronavirus type 2
COVID-19	Coronavirus disease 2019
LCI	Lung Clearance Index
WHO	World health organization
PA	Pseudomonas aeruginosa
S1RBD	receptor-binding domain of S1 subunit of spike protein
CF-LI	CF liver transplantation
CF-LU	CF lung transplantation
